# Global Distribution of Rubella Virus Genotypes

**DOI:** 10.3201/eid0912.030242

**Published:** 2003-12

**Authors:** Du-Ping Zheng, Teryl K. Frey, Joseph Icenogle, Shigetaka Katow, Emily S. Abernathy, Ki-Joon Song, Wen-Bo Xu, Vitaly Yarulin, R.G. Desjatskova, Yair Aboudy, Gisela Enders, Margaret Croxson

**Affiliations:** * Georgia State University, Atlanta, Georgia, USA; † Centers for Disease Control and Prevention, Atlanta, Georgia, USA; ‡ National Institute of Infectious Diseases, Tokyo, Japan; § Korea University, Seoul, Korea; ¶ Chinese Centers for Disease Control and Prevention, Beijing, China; # Institute of Viral Preparations, Moscow, Russia; ** Chaim Sheba Medical Center, Tel-Hashomer, Israel; †† Institute for Virology, Infectiology and Epidemiology, Stuttgart, Germany; ‡‡ Auckland Hospital, Auckland, New Zealand

**Keywords:** Rubella virus, virus genotypes, molecular epidemiology, virus distribution, virus recombination

## Abstract

Phylogenetic analysis of a collection of 103 E1 gene sequences from rubella viruses isolated from 17 countries from 1961 to 2000 confirmed the existence of at least two genotypes. Rubella genotype I (RGI) isolates, predominant in Europe, Japan, and the Western Hemisphere, segregated into discrete subgenotypes; intercontinental subgenotypes present in the 1960s and 1970s were replaced by geographically restricted subgenotypes after ~1980. Recently, active subgenotypes include one in the United States and Latin America, one in China, and a third that apparently originated in Asia and spread to Europe and North America, starting in 1997, indicating the recent emergence of an intercontinental subgenotype. A virus that potentially arose as a recombinant between two RGI subgenotypes was discovered. Rubella genotype II (RGII) showed greater genetic diversity than did RGI and may actually consist of multiple genotypes. RGII viruses were limited to Asia and Europe; RGI viruses were also present in most of the countries where RGII viruses were isolated.

Rubella virus infection during the first trimester of pregnancy can lead to severe birth defects (congenital rubella syndrome) (*1*). Live attenuated vaccines, available since the late 1960s (*2*), are currently in use in roughly half of the countries in the world, including all industrialized countries, although vaccine coverage varies widely (*3*). Concentration on comprehensive rubella vaccination has recently increased in developing countries in conjunction with measles elimination efforts, particularly in Latin America (*4*,*5*). As part of the surveillance component of these efforts, an understanding of the worldwide molecular epidemiology of rubella virus, which is limited, is necessary.

Rubella virus is an RNA virus that is the sole member of the *Rubivirus* genus, within the *Togaviridae* family (*6*). The rubella virus genome is ~10,000 nucleotides and encodes five protein products, including three virion proteins: the C or capsid protein and two envelope glycoproteins, E1 and E2. The E1 gene sequence has been used for genotyping and phylogenetic analysis of rubella virus isolates (*7*–*10*). Thus far, rubella viruses from Europe, Asia, and North America have been found for the most part to group in a single genotype (Rubella Genotype I or RGI) that has a maximum diversity at the nucleotide level of ~5%. However, a limited number of viruses from Asia (China and India), and more recently Italy, formed a distant phylogenetic branch, differing from RGI viruses by 8% to 10%, which was designated Rubella Genotype II (RGII) (*8*,*9*,*11*,*12*). These two genotypes belong to the single rubella virus serotype (*11*). Because of limited sampling, the geographic range of RGII has not been determined.

This study was designed to increase information and understanding on worldwide molecular epidemiology of rubella virus. We have performed combined phylogenetic analysis on viruses from earlier studies (*8*–*10*) and, to gain further information on RGII viruses, we included viruses collected from the Eastern Hemisphere, namely Russia, South Korea, China, New Zealand, and Israel.

## Materials and Methods

### Rubella Isolates and Sequences

A total of 103 rubella virus E1 gene nucleotide sequences were used in this study; “new” sequences not reported in previous studies are shown in Table 1, a complete list is available online (http://www.cdc.gov/ncidod/EID/vol09no12/03-0242.htm#table1). The length of the sequence was 1179 nt, which covered 8291–9469 nt of the rubella virus genome (the complete E1 gene is between nts 8258–9700 [*13*]). This collection consisted of sequences from new isolates from China, Israel, Japan, Korea, New Zealand, and Russia and representative sequences from previous studies (*8*–*10*). Methods of isolate propagation and E1 gene sequence determination were as previously described (*9*).

**Table 1:** “New” rubella virus E1 gene sequences used in this study^a^

Isolate	Isolation site and y	GenBank no.
China		
AH2/AH-CHN99	Anhui, China 1999	AY326350
AH5/AH-CHN99	Anhui, China 1999	AY326351
Germany		
BCM/-GER91	Germany 1991	AY326341
G696/-GER98	Germany 1998	AY326342
Israel		
I11/TA-ISR68	Tel Aviv, Israel 1968	AY326335
I19/HF-ISR72	Haifa, Israel 1972	AY326338
I9/JS-ISR75	Jerusalem, Israel 1975	AY326334
I13/BB-ISR79	Bene-Berak, Israel 1979	AY326336
I15/JF-ISR78	Jaffa, Israel 1978	AY326337
I34/TB-ISR88	Tiberias, Israel 1988	AY326339
I76/EV-ISR92	Ein-Vered, Israel 1992	AY326340
India		
MTS/-IND00	India 2000	AY326343^b^
Japan		
J05/TK-JPN93	Tokyo, Japan 1993	AB072382
J91/GM-JAP94	Gunma, Japan 1994	AB072384
J86/ST-JAP95	Saitama, Japan 1995	AB072387
J13/HS-JAP97	Hiroshima, Japan 1997	AY397695
Korea		
AN1/SO-KOR95	Seoul, Korea 1995	AY326345
AN3/SO-KOR96	Seoul, Korea 1996	AY326346
AN5/SO-KOR96	Seoul, Korea 1996	AY326347
AN6/SO-KOR96	Seoul, Korea 1996	AY326348
New Zealand		
JC1/AL-NEZ81	Auckland, NZ 1981	AY326331
JC2/AL-NEZ91	Auckland, NZ 1991	AY326332
JC5C/AL-NEZ91	Auckland, NZ 1991	AY326333
Russia		
C4/MO-RUS67	Moscow, Russia 1967	AY247015
C19/MO-RUS68	Moscow, Russia 1968	AY247016
C44/MO-RUS69	Moscow, Russia 1969	AY247017
C68/MO-RUS73	Moscow, Russia 1973	AY247018
C74/MO-RUS97	Moscow, Russia 1997	AY247019

^a^New E1 gene sequences not previously reported in earlier studies (8-12) are listed in this Table. A complete list of sequences used in this study is available online (http://www.cdc.gov/ncidod/EID/vol09no12/03-0242.htm#table1) ^b^Isolated in Seattle, WA, USA

### Genetic Sequence Similarities and Distances

Distance matrix tables were computed by using the Olddistance with Simple correction method in the GCG software package (Wisconsin package version 10.0, Genetics Computer Group, Madison, WI). These tables were used to calculate the average genetic distance among viruses in a single genotypic group or between viruses in two genotypic groups.

### Phylogenetic Analysis

**Figure 1:**
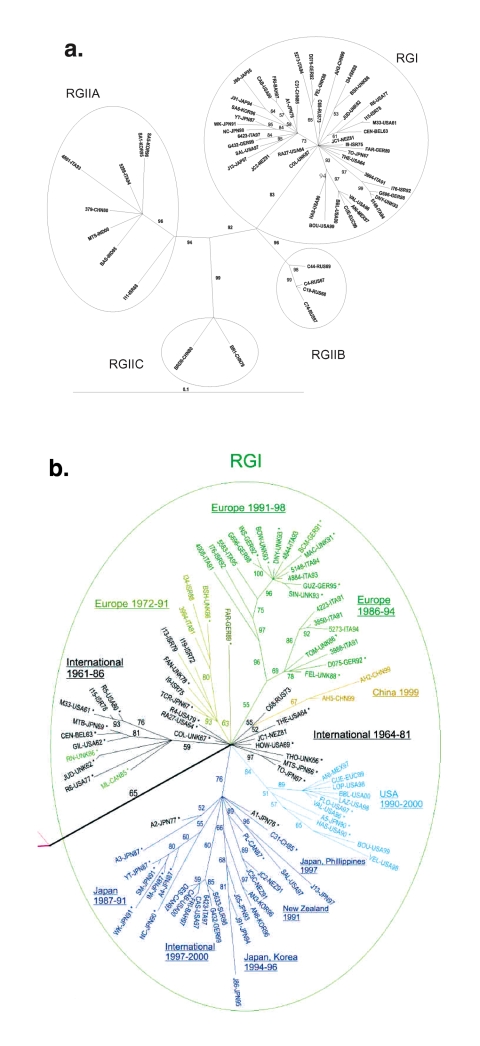
Phylogenetic trees. Unrooted tree was made by the maximum likelihood method in the Tree-Puzzle 5.0 program (25,000 puzzling steps for the tree in A; 10,000 puzzling steps for the tree in B) using the complete E1 gene sequence (1179 nt). Bootstrapping values (out of 100) for each node are given. The tree in A was constructed with half of the rubella genotype I (RGI) and all of the RGII sequences (to allow the reader to read the RGI virus designations); the tree in B is a blowup of the RGI node from a tree constructed with all of the sequences. In B, sequences used in the previous study (*8*) are designated by an (*), and sequences of viruses isolated before 1980 are in black. Branches are color-coded as follows: RGI Intercontinental (International) 1961–1986 and 1964–1981, black; RGI Europe 1972–1991, gold; RGI Europe 1986–1994 and Europe 1991–1998, green; RGI China, 1999, gold; RGI USA, 1990–2000, light blue; and branch containing sub-branches from Japan 1987–1991, Intercontinental (International) 1997–2000, Japan, Korea 1994–1996, New Zealand, 1991, and Japan-Philippines, 1997, dark blue. Of these, the black Intercontinental (International), green Europe, light-blue USA, and dark-blue branches were recognized in the previous study (the light-blue branch as US-Japan and the dark-blue branch as Japan-Hong Kong).

**Figure 2:**
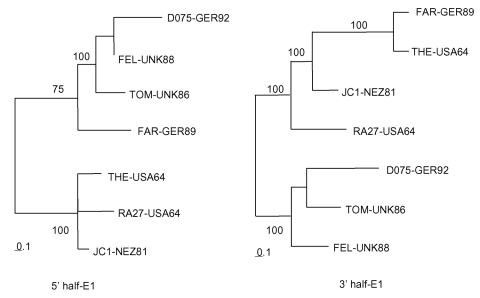
Phylogenetic trees. Trees were made by the maximum likelihood method in the Tree-Puzzle 5.0 program (1,000 puzzling steps) using the 5′ half (640 nt: 8291–8930) or 3′ half (539 nt: 8931–9469) of the E1 gene sequence. Bootstrapping values (out of 100) for each node are given.

The phylogenetic trees (Figures 1 and 2) were made using Tree-Puzzle 5.0 (*14*) (maximum likelihood [ML] criterion with 10,000 or 25,000 quartet puzzling steps and HKY85 model of substitution [*15*]) and viewed by using TreeView (*16*). To test the consistency of branching, additional software packages were used to construct trees, including PAUP (David L Swofford. 2001. ver 4.0β, Sinauer Associates, Sunderland, MA) and PHYLIP (Joseph Felsenstein, ver 3.6α, July 2000, WA). Both ML and maximum parsimony (MP) algorithms in these packages were used. A histogram of the ML distances computed by Tree Puzzle 5.0 (*14*) was constructed with Excel (Microsoft, Redman, WA).

## Results

The sequences used in this study consist of representative sequences from three earlier studies (Europe, North America, and Asia, 1961–1997 [8]; Italy, 1991–1997 [9]; and the United States, 1997–2001 [10]) and sequences of viruses collected in Russia, South Korea, New Zealand, Israel, China, Japan, and the United States that have not been reported (these new sequences are listed in Table 1; a complete sequence list is available online (http://www.cdc.gov/ncidod/EID/vol09no12/03-0242.htm#table1). The sequences from the three earlier studies have not previously been amalgamated into a single phylogenetic study. Phylogenetic trees produced with different programs yielded the same major branches, and only representative trees are shown (Figure 1). Branches were defined as reproducible clusterings of sequences by these different programs.

Most of the new sequences belonged to RGI, which emanate from a single node in the tree shown in Figure 1. For simplicity, the tree shown in Figure 1A contains only half of the RGI sequences; the RGI node with all of the RGI sequences is shown in Figure 1B. New sequences belonging to RGI included both from China, all three from New Zealand, all four from Japan, five of six from Israel, two of four from South Korea, and one of five from Russia. Additionally, all of the sequences from the U.S. study (*10*) and 19 of 21 from the Italy study (*9*) were RGI. Fourteen sequences were grouped on branches removed from the RGI node, including the three viruses originally used to define RGII, two RGII viruses isolated from Italy (1994) [9], and 9 of the new sequences: 4 from Russia (3 from 1967 to 1969 and 1 from 1997), 2 from South Korea (1996), 1 from Israel (1968), 1 from China (1979), and 1 isolated in the United States from a case contracted in India (2000).

### RGI Viruses

The sequences of viruses isolated before 1980 are colored black in the expanded RGI node (Figure 1B). These sequences, which were from viruses isolated in Europe, North America, and Asia, congregated around the central node (International 1964–1981) or segregated into one of two branches, denoted International 1961–1986 and Europe 1972–1991. Many of these sequences were used in our previous study (*8*; marked with an asterisk) in which the sequences in the International 1961–1986 branch formed three related branches, the sequences in the International 1964–1981 nodal congregation were spread along the baseline of the dendogram, and the sequences in the Europe 1972–1991 branch were at the base of the Europe branch. Additions to the International 1964–1981 nodal congregation included sequences from a virus isolated in Russia in 1973 and a virus isolated in New Zealand in 1981; a sequence from a virus from Israel 1978 was added to the International 1961–1986 branch. The Europe 1972–1991 branch (colored gold in Figure 1) contained viruses from Israel (1972, 1975, 1979, and 1988), Italy (1991), and the United Kingdom (1978 and 1986; both used in the previous study [8]). The TCRB vaccine strain (Japan, 1967) sequence segregated onto this branch; in our previous study (*8*); the TCRB sequence was at the base of the European branch. In summary, while the branching pattern of sequences of viruses isolated internationally before ~1980 (*8*) was basically preserved, the addition of sequences led to definition of a new branch of viruses from Europe and Israel, isolated between 1972 and 1991.

Newly added sequences of viruses isolated after 1990 segregated primarily onto the three continent-restricted branches defined in our earlier study (*8*), generally in a geographically consistent manner. These branches, designated Europe, Japan-Hong Kong, and US-Japan in the earlier study (*8*), are colored green, dark blue, and light blue (the US-Japan branch was redesignated USA 1990–2000; see below). The Europe branch was augmented by sequences of viruses from Italy, Germany, and Israel (1991–1998) and subdivided into two sub-branches, one containing sequences of viruses isolated in Europe from 1986 to 1994 (Europe 1986–1994) and a second containing sequences of viruses isolated in Europe and Israel from 1991 to 1995 (Europe 1991–1998). Several newly added sequences of viruses from the United States and Latin America (1997–2000) enlarged the USA 1990–2000 branch; these viruses are thought to be endemic in regions bordering the United States and to seed rubella outbreaks and clusters in that country (*10*). The only two viruses from Japan in this branch were isolated from a single province in 1991; it was previously hypothesized that this finding represented transport of viruses from the United States to Japan or vice versa (*8*). In light of the isolation of several viruses in this group subsequently from the United States or Latin America and no viruses from Japan (either before or after 1991), the former transport pathway likely occurred; thus, this branch has been redesignated USA 1990–2000.

The Japan-Hong Kong branch expanded considerably to include sequences of a pair of viruses from Japan and the Philippines (1997) that were related to each other, two sequences of viruses from New Zealand (1991) that were related to each other, and sequences of a group of five related viruses isolated in Japan and South Korea from 1995 to 1996. Another distinct sub-branch was formed by a closely related group of sequences of viruses associated with outbreaks in Europe, the United States, and the Caribbean from 1997 to 2000 (Intercontinental, 1996–2000). The viruses in this branch caused several outbreaks in the northeastern United States and on cruise ships in Florida (*10*) and were isolated during a rubella epidemic in Italy in 1997 (*9*); the relatedness of these viruses was not previously recognized. A novel branch contained sequences of two viruses from China (1999) (China, 1999, gold in Figure 1B).

### RGII Viruses

The tree depicted in Figure 1A indicated that sequence divergence was greater among RGII viruses than among RGI viruses. RGII viruses segregated into three distinct clusters (RGIIA, RGIIB, and RGIIC), all of which were supported by bootstrapping values of >90. Only the RGIIA cluster contained more than one virus isolated within the past decade, and this active cluster contained viruses from diverse locations (India, China, Korea, Italy). The other two clusters contained viruses from a single location (RGIIB-Russia; RGIIC-Beijing, China). The RGII clusters were more distant from each other than were the RGI branches. Variability between RGI and the three RGII clusters is shown in Table 2. Maximal variability among RGI viruses was ~5.8%, however, RGII viruses varied by up to ~8.0%. Viruses within each of the three RGII clusters varied by up to 5.5% and the clusters differed from each other by an average of ~7% (range of variability between viruses in the RGII clusters: RGIIA-RGIIB: 6.35%-7.78%; RGIIA-RGIIC: 6.86%-7.95%; RGIIB-RGIIC: 6.85%-7.53%). Average variation of the RGII clusters from RGI was from ~6% to 8% (range 5.5%-10.3%, which translates to a range of 0.8% to 2.1% variation at the amino acid level).

**Table 2:** Intra- and intergoup genetic distances among rubella genotype I (RGI) and RGII clusters^a^

Genotype/cluster	Intragroup variability	Mean distance from			
		RGII	RGIIA	RGIIB	RGIIC
RGI	0.08–5.75	7.28	7.59	6.20	8.21
RGII	0–7.95				
RGIIA	0–5.41			7.24	7.13
RGIIB	0.42–1.95				7.19
RGIIC	2.54				

^a^Ranges and mean genetic distances (% nucleotide difference) was determined from all pairwise combinations from viruses in these groups (Figure 1).

### Evidence for Recombinant Virus

Use of the entire E1 gene resulted in excessive time being required to run phylogenetic programs with the large number of sequences included in this study (e.g., trees in Figure 1 took days to compute). We therefore investigated using smaller segments within the E1 gene (five windows of ~400 nt encompassing nt 8291–8640, 8491–8890, 8687–9088, 8891–9290, and 9100–9469 of the genome) to construct phylogenetic trees. All major branches shown in Figure 1 were preserved in trees constructed from each of these windows, although placement and joining of the branches varied at the base of the tree. During this investigation, the sequence of one isolate, FAR-GER89, was found to group with three contemporaneous viruses in the Europe 1986-1994 branch (TOM-UK86, FEL-UK88, and D075-GER92) in the three 5′-most windows within the E1 gene, but with three International 1964–1981 viruses (RA27-USA64, JC1-NEZ81, and THE-USA64) in the two 3′-most windows (illustrated in Figure 2). When these seven sequences were aligned, over the 5′ 630 nt, 14 nt characteristics of the TOMI/FEL/D075 or THE/JC1/RA27 sequences were identified; of these, FAR had 12 characteristics to the TOMI/FEL/D075 sequences, 1 characteristic to the THE/JC1/RA27, sequences and 1 unique nucleotide (data not shown). In contrast, over the 3′ 540 nt of the E1 gene, 11 nt characteristics of the TOM/FEL/D075 or THE sequence were identified and of these 11 characteristic nts, FAR shared all 11 with the THE sequence and none with the TOM/FEL/D075 sequences. These results indicate that FAR may be a recombinant between viruses from these two Genotype I groups. The FAR-GER89 sequence is on its own branch, emanating from the RGI node (Figure 1); similar observations have been made with poliovirus recombinants (*17*).

## Discussion

In this study, we extended phylogenetic analysis of rubella viruses collected worldwide. The baseline for this study was an analysis (*8*) of viruses collected from Europe, North America, and Asia, 1961–1997; we found that an intercontinental genotypic group existed until ~1980 and was replaced by continent-restricted genotypic groups after ~1980. In this study, this analysis was augmented by inclusion of comprehensive collections from Italy (*9*) and the United States (*10*) as well as viruses collected from new locations. In the previous analysis (*8*), a second genotype was identified among a limited number of specimens collected from Asia and a specific goal of this study was to analyze viruses from new locations in hopes of learning more about this second genotype.

Use of the entire E1 gene resulted in excessive time being required to run phylogenetic programs with the large number of sequences included in this study. We therefore investigated using ~400-nt segments within the E1 gene. While the major genotypic and subgenotypic grouping were preserved in these segmental trees, the trees produced from the nt 8687–9088 window were most similar to those produced from the entire E1 gene. This window was named the “molecular epidemiology window.” As shown in Table 3, this window had a similar to somewhat higher intersequence variability than the E1 gene and preserved the GC content and intersequence transition/transversion ratio exhibited by the E1 gene. Although virus isolates were used in this study, amplification and sequencing of E1 gene segments directly from clinical specimens using reverse transcription-polymerase chain reaction (RT-PCR) are at hand (*18*–*22*). Our findings indicate that RT-PCR products from any region of the E1 gene will produce phylogenetic trees consistent with those from the entire E1 gene. However, thus far the regions amplified by in these studies have not been standardized.

**Table 3:** Comparison of genotypic statistics using the E1 gene and the molecular epidemiology window (MEW)

Window	G+C content^a^	Transition/transversion^a^	Intergenotypic distance^a,b^			Intragenotypic distances^a,c^				
			Range	Mean	Mean RGI vs RGII	RGI		RGII		
						Range	Mean	Range	Mean	
E1	66.6	6.34	0.08~10.32	4.92	7.28	0.08~5.75	3.55	0~7.95	5.66	
MEW	66.1	6.15	0~11.69	4.97	8.32	0~5.97	3.49	0~8.71	6.61	

^a^Statistics were determined from all sequences listed in Table 1. ^b^The range and mean of genetic distances (% difference) were determined by using all sequences. The mean rubella genotype I (RGI) vs. RGII was determined from all of the pairwise RGI-RGII combinations. ^c^The range and mean of genetic distances were determined for RGI and RGII viruses separately.

Analysis of the segmental trees indicated that the FAR-GER89 isolate might have arisen by a recombination event within the E1 gene between a virus from the Europe 1986–1994 branch (TOM-UK86, FEL-UK88, D075-GER92) and a virus from the International 1964–1981 nodal cluster (THE-US64, RA27-USA64, JC1-NEZ81). Such an event is temporally consistent because viruses related to the International 1964–1981 nodal cluster have been isolated in Italy as late as 1991 (*9*) but were not included in this study. Although rubella virus recombination in cell culture has been documented (*23*), this report is the first of a natural recombinant. Although the FAR-GER89 isolate was related to the RA27/3 vaccine strain in the 3′ end of the E1 gene, the FAR-GER89 sequence contains none of the nucleotides characteristic of the RA27/3 sequence (*7*); thus, the recombination event did not involve a vaccine virus.

In our previous analysis (*8*), all of the viruses isolated in Europe, North America, and Japan were RGI. This finding was maintained with the expanded collections from all three regions used in this study with the exception of two RGII viruses isolated in Italy. Additionally, most of the viruses in the expanded RGI collection from these regions segued into the previously defined RGI branches (*8*), with the exception of a newly defined Europe 1972–1991 branch that contained viruses at the base of the Europe branch defined in the previous study and a novel branch consisting of two viruses from China (1999). In the case of viruses from Europe, with a larger number of viruses representing a longer time span, these viruses belonged to two branches, the second of which divided into two sub-branches. The RGI viruses from Israel were related to the European viruses and fit into the temporal pattern of isolation of the European viruses. The temporal pattern of isolation of viruses in the European branches and sub-branches (1972–1991, 1986–1994, and 1991–1998) indicated that temporal displacement of genotypic groups occurred, as had been noted in our previous study on viruses from Italy (*9*). These European branches and sub-branches have been more recently displaced by the International 1997–2000 sub-branch of the Japan-Hong Kong branch. The addition of viruses isolated recently in the United States (*10*) clarified that the US-Japan branch in the previous study (*8*) was a United States branch, viruses from which, when transported to Japan, caused an outbreak in 1991. Viruses in the USA 1990–2000 branch were related to the limited number of viruses available from Latin America.

The branch termed a Japan-Hong Kong branch in our earlier study (*8*) contained viruses from Japan and Hong Kong isolated between 1976 and 1991. Ten representative viruses were selected from that branch for this study, including 2 viruses from Japan (1976 and 1977) and 1 virus from Hong Kong (1985) that grouped at the base of the branch as well as several viruses from Japan (1987–1991) that formed a sub-branch. This branch was expanded by the addition of four sub-branches containing viruses from New Zealand, 1991; Japan and Korea, 1994–1996; Japan and the Philippines, 1997; and an international sub-branch that contained viruses associated with epidemics and outbreaks in Europe and the United States during 1997 to 2000 that were unrelated to viruses previously isolated from those regions. Recent evidence indicates that these viruses are closely related to viruses from China (Z. Zhen et al., unpub. data). Thus, the appearance of this sub-branch in Europe and the United States was likely due to intercontinental transport. Considering that viruses from this sub-branch have been predominant in Europe since 1997 and have been one of the two genotypic groups isolated recently in the United States (USA 1990–2000 is the other), an international genotypic branch of rubella virus appears to have emerged after a ~20-year hiatus since the previous demonstrable international branch. Along with this branch, the two other currently active branches are the USA 1990–2000 branch and the China 1999 branch, only represented by two viruses from the collection used in this study.

**Figure 3:**
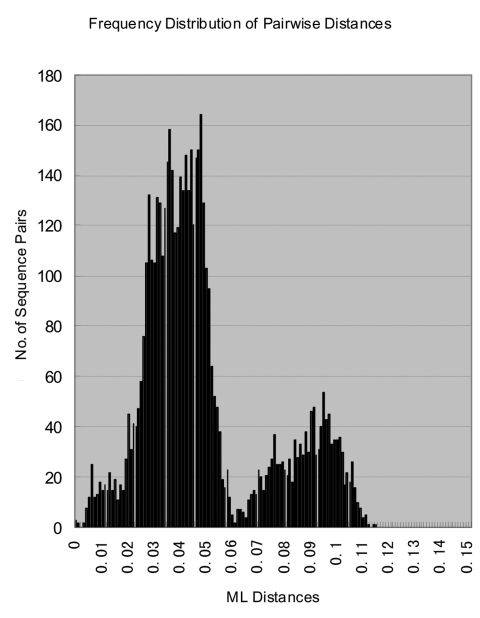
Histogram of genetic distances between rubella virus sequences. The histogram, showing the distribution of all of the pairwise distances between the rubella virus sequences in the study, was constructed from the maximum likelihood distance matrix computed by Tree Puzzle 5.0 program.

With its expanded virus collection, this study demonstrates that RGI rubella virus isolates segregate into discrete subgenotypic groups (i.e., branches) that exhibit geographic and temporal consistency. These groups could provide the basis for a standard classification scheme, as has been developed for other viruses. In this study, the number of available RGII viruses also increased to 14 in contrast to the 3 available in the previous analysis; the RGII viruses segregated into three discrete clusters (RGIIA, RGIIB, and RGIIC). RGI viruses formed a discrete cluster clearly distinguishable from the most closely related RGII cluster (RGIIB) with no indication of intermediate viruses. The maximum diversity within RGI was 5.8%, and RGI viruses were an average of >7% different from the RGII viruses (Table 2); when one considers the extensive nature of the RGI virus collection, this is a working definition of a rubella virus genotype. RGII was originally defined to contain a limited number of sequences (three) that distinctly differed from the RGI viruses that made up most of the collection (*8*). The sequences in each of the RGII clusters vary by up to 5.4%, and the average distance between viruses in each of the clusters is >7% (Table 2). Thus, each of these clusters could represent a genotype. This hypothesis is strengthened by a histogram of the intersequence distances that show a bimodal distribution with peaks from 0.5%-6.0% to 6.5%-11.0%, corresponding to intra- and intergenotypic distances (Figure 3). The bimodal distribution also indicated equivalency of genotypic groups (*24*). However, in light of the limited number of RGII specimens, more specimens are needed to fully characterize the extent of the diversity within non-RGI viruses, and criteria need to be established for definition of additional genotypes. In this regard, as represented by the viruses in this collection, only one of the RGII clusters (RGIIA) has been repeatedly active in the past decade. However, viruses belonging to the RGII C cluster (China) have been recently isolated (Z. Zhen et al., unpub. data).

**Figure 4:**
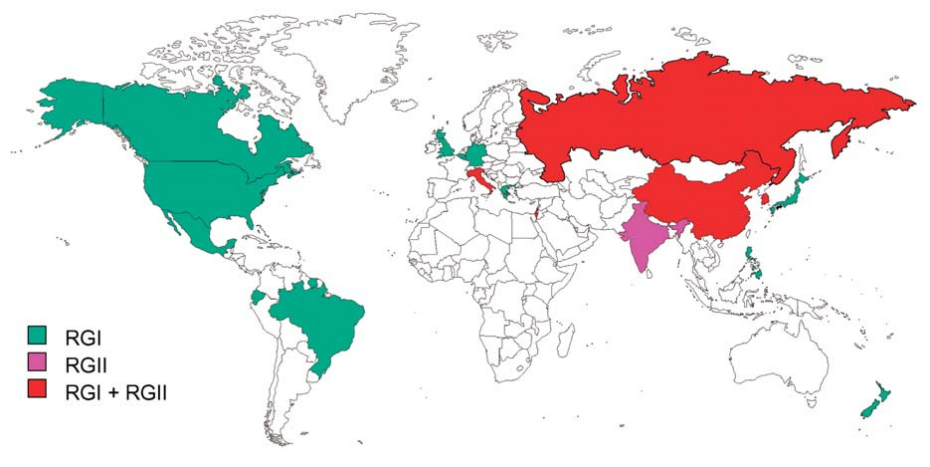
Distribution of rubella genotypes.

Figure 4 shows the countries from which RGI and RGII viruses have been isolated. RGII has not been isolated from an indigenous case outside of the Eastern Hemisphere. Only RGI viruses have been isolated from the United Kingdom, Belgium, Germany, Japan, and New Zealand; other studies have shown RGI viruses in Brazil (*10*) and Greece (*23*). Only RGII viruses have been isolated from India, and both genotypes have been isolated from Russia, Italy, Israel, China, and Korea. In Italy and Korea, the two genotypes were isolated in the same year. In both Italy and Israel, isolation of RGII viruses was only during a single year. By contrast, RGI viruses were isolated in both previous and subsequent years. This finding indicates that these RGII viruses were imports, although the relative distant relatedness of the two Italy isolates suggests at least two importation events, albeit in a single year (*9*). In China, RGII viruses were isolated in 1979 and 1980; the most recent isolates (1999) were RGI, although RGIIC viruses were recently isolated (Z. Zhen et al., unpub. data). These data indicate that RGI has a wider worldwide distribution than does RGII and that in much of the world RGI is the sole genotype. Recent RGII activity is confined to Asia and overlaps with RGI; however, the dataset from areas in which RGII viruses appear to circulate is limited. Additionally, as shown in Figure 4, large regions of the world remain to be sampled to complete the rubella virus genotypic picture.
